# Transcriptomic Profiling of Cutaneous Melanoma Metastases Treated With Microwave Ablation—Pilot Study

**DOI:** 10.1111/cts.70638

**Published:** 2026-06-18

**Authors:** Purevsuren Losol, Daniel O'Driscoll, Andres Vallejo Pulido, Adrian von Witzleben, Konstantinos Boukas, Matthew Sommerlad, Christian Ottensmeier, Michael R. Ardern‐Jones

**Affiliations:** ^1^ Clinical Experimental Sciences, Faculty of Medicine University of Southampton Southampton UK; ^2^ Department of Dermatology University Hospital Southampton NHS Foundation Trust Southampton UK; ^3^ Department of Dermatology Imperial College Healthcare NHS Trust London UK; ^4^ Department of Otorhinolaryngology, Head and Neck Surgery University of Ulm Ulm Germany; ^5^ Wessex Investigational Sciences Hub, Cancer Sciences, Faculty of Medicine University of Southampton, Southampton General Hospital Southampton UK; ^6^ Department of Histopathology University Hospital Southampton NHS Foundation Trust Southampton UK; ^7^ Faculty of Medicine Liverpool Head and Neck Center, Institute of Systems, Molecular and Integrative Biology, University of Liverpool Liverpool UK

**Keywords:** melanoma, microwave, transcriptomics, treatment

## Abstract

Microwave ablation induces heating of tissue. High energy results in thermal cell death, but low energy treatment without tissue necrosis has been shown to induce cutaneous immunity in an HPV wart model. It is approved as an effective cancer treatment for solid organ cancers, and therefore it is of interest to know if microwave delivered directly to the skin holds potential for treatment of skin cancer. This pilot study focused on transcriptomic profiling of cutaneous melanoma metastases to investigate molecular changes associated with microwave therapy. Seven adult patients with skin metastases from malignant cutaneous melanoma, not resolving on standard treatment, were recruited. Microwave energy was applied to separate melanoma metastases. Morphological, histological, and transcriptomic changes assessed via tissue RNA sequencing were evaluated. Three participants showed complete response, while four showed partial response by histological assessment. In complete responders, skin lesion RNA sequencing after treatment, compared with baseline, identified increased inflammation (*CXCL5, CXCL8*, *IL1A*, *COL1A1*) and downregulated cancer markers (*PRAME*, *S100B*, *MLANA*, *STK32A*). Compared with partial responders, complete responders showed enrichment of *FABP4* and reduced expression of cancer markers. Microwave therapy produced local tumor responses and associated inflammatory transcriptomic changes in complete responders, supporting further clinical evaluation in cutaneous melanoma metastases.

## Study Highlights

- What Is the Current Knowledge on the Topic?
  ○ Microwave ablation induces thermal cell death and has demonstrated potential both in stimulating skin immune responses and in treating internal organ cancers. However, its effects on skin cancers, particularly cutaneous melanoma metastases, remain largely unexplored.
- What Question Did This Study Address?
  ○ This proof‐of‐concept study investigated whether directly delivered microwave therapy could safely reduce tumor volume and affect tumor biology in cutaneous melanoma metastases.
- What Does This Study Add to Our Knowledge?
  ○ Microwave treatment over 3 weeks reduced tumor volume in treated lesions. Complete clearance of melanoma was observed in 3/7 cases, accompanied by decreases in cancer‐associated markers.
- How Might This Change Clinical Pharmacology or Translational Science?
  ○ The findings suggest microwave therapy could provide an alternative treatment option for cutaneous melanoma metastases, particularly when immunotherapy fails or surgery is not feasible. Observed tumor reduction, complete clearance in some lesions, and molecular evidence of increased inflammation and decreased tumor activity support further investigation of its potential clinical and translational applications.

## 1 Introduction

Cutaneous melanoma is a malignant tumor arising from melanocytes, and historically, outcomes for those with advanced (stage III/IV) disease have been extremely poor; the five‐year survival rate for untreated stage IV melanoma has been reported to be less than 10% [1]. Immune checkpoint inhibitor therapy for melanoma has led to a revolution in treatment, with 5‐year survival on combination therapy exceeding 50% [2]. Nonetheless, half of patients do not respond to immunotherapy and therefore additional treatment strategies are needed [3]. Among these, electrochemotherapy with bleomycin or cisplatin achieves high local response rates (approximately 77%) with low toxicity [4, 5], making it effective for accessible cutaneous lesions. Similarly, talimogene laherparepvec (T‐VEC) and intralesional interleukin‐2 (IL‐2) offer high local response rates and minimal systemic side effects [6, 7, 8].

Beyond direct cytotoxicity, thermal ablation modalities such as cryoablation and radiofrequency ablation have been shown to induce necrotic cell death, releasing tumor antigens and damage‐associated molecular patterns that activate dendritic cells and prime tumor‐specific T‐cell responses [9]. Microwaves (30 MHz to 30 GHz) exist in the electromagnetic spectrum between radiofrequency and visible light and have been widely used as a means for delivering heat energy to induce thermal ablation in the treatment of cancer, especially for inoperable liver tumours [10]. We have previously shown keratinocytes treated with microwaves can prime dendritic cells to enhance cross‐presentation of HPV antigens to CD8^+^ lymphocytes and effectively treat viral warts, raising the possibility of microwave‐induced HPV immunity [11]. The same modality has demonstrated efficacy in the treatment of actinic keratoses [12]. We hypothesized that microwave treatment may alter the tumor immune microenvironment and stimulate an adaptive immune response. This study therefore aimed to investigate the molecular effects of directly delivered microwave therapy on cutaneous melanoma metastases using transcriptomic analyses.

## 2 Methods

This single‐arm, non‐randomized proof‐of‐concept study was carried out at University Hospital Southampton, UK, between January 2019 and March 2023 (National research ethics approval 17/SC/0287). All participants provided written informed consent. Participants were eligible if they were over 18 years old, had stage IV or inoperable stage III melanoma, and were deemed either unwilling or unsuitable to undergo alternative metastatic treatment options. Exclusion criteria comprised age under 18 years and inability to provide informed consent. There was no attrition in this study. The Swift microwave device (Emblation Medical Ltd., UK) designed to deliver targeted microwave energy through a probe applicator was employed. The microwave device was initially developed for plantar warts where treatment is administered with incremental titration, as tolerated, using either higher energy (30–50 J) or lower energy (10–20 J), delivered at a power setting of 10 W for 5 s [11, 13]. Different energy levels were used to assess whether treatment intensity influences tumor response.

At day 0 (V1), participants received a baseline assessment, and skin metastasis volume was estimated by measuring width, length, and elevation above the skin surface with a digital vernier caliper (Louisware). Three co‐located metastases were selected for high energy (T1), low energy (T2) microwave, or untreated control (C). Punch biopsies of lesion T1 were performed under local anesthetic pre‐ and post‐treatment for histological analysis and RNA sequencing. All V1 tumor volume measurements were taken post biopsy in T1 lesions, whereas T2 and control lesions were not biopsied. Lesions < 7 mm probe diameter were treated with a single application, whereas larger lesions required multiple applications to ensure full coverage. Participants were reviewed at weeks 1, 2, and 3, and a re‐biopsy of T1 was obtained at visit 4. No changes were made to standard oncology care. The primary endpoint was response in the treated nodule, assessed histologically at T1. Complete response indicated no viable melanoma, and partial response indicated residual tumor.

Tumor biopsies were removed and fixed in 10% neutral‐buffered formalin before paraffin processing. Serial 4 μm sections were cut and one slide of each sample was stained with hematoxylin and eosin (H&E). All samples were reviewed and assessed microscopically by a pathologist (MS). To analyze RNA expression, regions of interest (ROI) were marked on H&E‐stained samples and microdissected from a subsequent fresh, unstained 5 μm cut mounted on a glass slide.

Targeted RNA sequencing of 1392 transcripts, focused on tumor/immune interactions, with HTG EdgeSeq system's Precision Immuno‐Oncology Panel (PIP) was performed on formalin‐fixed paraffin‐embedded (FFPE) tissue for all samples [14]. Comparison of the expression profile of lesional tissue was made between pre‐treatment and post‐treatment skin biopsy samples. Detailed description of sample processing and library preparation have been previously reported [15]. The libraries were quantified using the KAPA Library Quantification Kit designed for Illumina Platforms (Roche Sequencing and Life Science, KAPA Biosystems, Wilmington, MA). The quantified libraries were normalized, equimolarly pooled at a concentration of 3 pM, and loaded on the Illumina NextSeq500 for single‐read deep sequencing.

The sequencing base calls were transformed into FASTQ and demultiplexed using module bcl2fastq2 v2.18 on IRIDIS HPS (UoS) with the option “‐barcode‐mismatches 0” [15]. Following this, FASTQ files were parsed on HTG Edge parser software and generated a gene expression count matrix. The RNA‐sequencing raw counts underwent processing using EdgeR, a Bioconducter package, on R v4.3.2, and normalization of the size factor was performed using Trimmed Mean of M‐values (TMM). Batch effect was corrected to reduce the bias generated across batches. Heatmaps were generated using the gplots package, while principal component analysis (PCA) was performed via singular value decomposition (SVD) and projected using base R functions. Gene expression results were corrected for multiple testing using the Benjamini‐Hochberg procedure and differentially expressed genes (DEGs) were considered significant only with a false discovery rate (FDR) < 0.05. Graphs were generated with GraphPad Prism software v10.2.1. The data underlying this article are available in the Gene Expression Omnibus (GEO) with accession number GSE279418.

## 3 Results

A total of 7 patients (3 male) were recruited, with a mean age of 70 ± 6 years (Table S1). All patients were assessed by the melanoma oncology multidisciplinary team to have stable melanoma disease (stable total body tumor volume as measured by CT) following advanced therapies but suffered ongoing symptomatic cutaneous melanoma metastases diagnosed clinically. All patients had three co‐located cutaneous metastases, one selected for each of the three study arms: control (C), high energy (T1), low energy (T2) microwave. T1 metastasis was biopsied pre‐treatment and post‐treatment, and histological analysis defined responder status.

**FIGURE 1:**
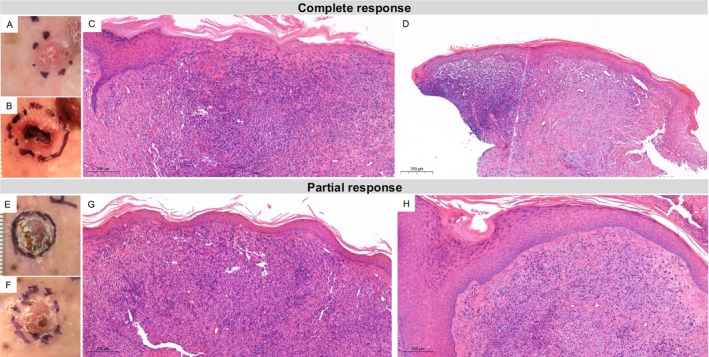
Representative clinical and histological images from complete and partial responders pre‐ and post‐treatment. Clinical image of a complete responder pre‐treatment (A) and post‐treatment (B). Representative high power (100 x) hematoxylin and eosin (H&E) image from the complete responder pre‐treatment, showing heavily inflamed skin with scattered atypical epithelioid cells in keeping with metastatic melanoma pre‐treatment (C), and post‐treatment, showing fibrosis and dense lymphohistiocytic inflammation but no evidence of residual melanoma (D). Clinical image of partial responder pre‐treatment (E) and post‐treatment (F). H&E image from the partial responder pre‐treatment, showing sheets of atypical epithelioid cells in keeping with metastatic melanoma (F), and post‐treatment, showing chronically inflamed skin with scattered atypical epithelioid cells in keeping with residual melanoma (H).

The clinical findings from this study have been previously reported [16]. After treatment, all T1 and T2 lesions showed a significant reduction in tumor volume post‐treatment. Lesions from the high energy treated metastases (T1) were re‐biopsied after treatment and response to microwave was verified by histological assessment (Figure 1). Using this rigorous classification, in the high energy nodule, three participants achieved a complete response, while four participants showed a partial response. All participants tolerated high energy microwave therapy under local anesthetic, while tolerability of lower energy treatment without anesthetic was more variable.

**FIGURE 2:**
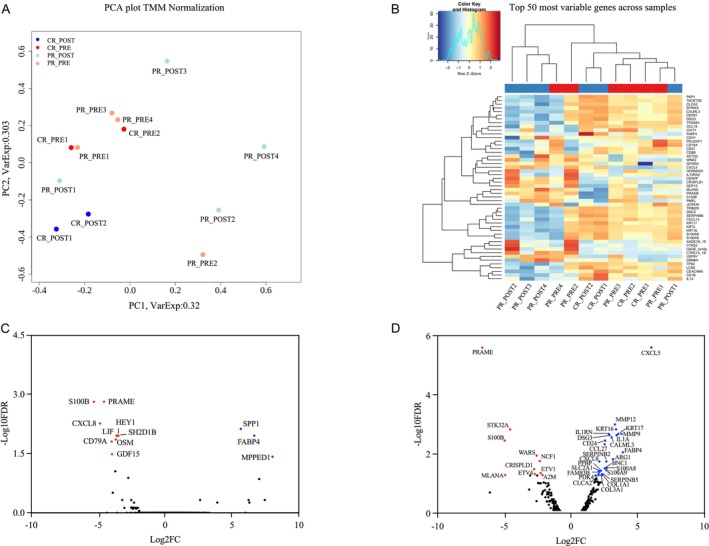
Transcriptional profiling of samples from complete and partial responders to microwave treatment in cutaneous melanoma. Principal component analysis (PCA) plot of all samples (A). Heatmap of all samples showing top 50 most variable gene expression patterns (B). Volcano plot of post‐treatment RNA‐seq data comparing complete responders and partial responders (C). Volcano plot of RNA‐seq data from complete responders comparing pre‐ and post‐treatment samples (D). Red dots represent downregulated genes, while blue dots indicate upregulated genes (FDR *p*‐value < 0.05), *N* = 6. CR, complete response, PR, partial response.

Patient's tumor biopsies were analyzed to assess differential gene expression between pre‐ and post‐treatment samples (Figure 2). One sample (complete response) was excluded from analysis as it failed both quality control (QC) and interquartile range (IQR) assessments. PCA and heatmap analyses demonstrated overall sample clustering and variable gene expression patterns across all samples, supporting transcriptional differences between complete and partial responders following microwave treatment (Figure 2A,B). Comparing post‐treatment transcriptomic signatures in complete and partial responders revealed a total of 3 upregulated and 9 downregulated differentially expressed genes (FDR *p*‐value < 0.05, Figure 2C, Figure S1, Table S2). Gene involved in metabolic and tissue remodeling processes, such as *FABP4* (fold change 6.66), was significantly upregulated following treatment. This was accompanied by the suppression of melanoma‐specific markers and cancer‐promoting genes, including *S100B* (fold change −5.31), *PRAME* (fold change −4.56), and *GDF15* (fold change −3.96). Notably, a subset of DEGs within this comparison was driven by a single patient (partial response), whose biopsy exhibited chronic inflammation with scattered atypical epithelioid cells in keeping with residual melanoma (Figure S1). Paired samples were calculated using pair‐analysis pre‐ and post‐treatment. In complete responders only, the comparison of samples pre‐ and post‐treatment detected 26 upregulated and 10 downregulated transcripts (Figure 2D, Table S3). The most highly upregulated transcripts included pro‐inflammatory chemokines and cytokines (*CXCL5* with fold change 6.03, *CXCL8* 2.13, *CCL27* 2.53, *IL1A* 3.42), as well as genes involved in skin cell differentiation (*KRT16* 3.38, *KRT17* 3.55) and collagen genes (*COL1A1* 2.35, *COL3A1* 2.28). Microwave treatment also resulted in the downregulation of prominent cancer markers in complete responders (*PRAME* −6.64, *MLANA* − 4.93, and *S100B* −4.96).

A heatmap displaying mean expression levels for differentially expressed genes across study groups illustrates distinct transcriptional profiles associated with treatment status (Figure S2).

## 4 Discussion

This pilot study set out to explore the transcriptomic profile of microwave treatment for cutaneous metastases, as this treatment modality has not been applied to cutaneous melanoma before. As typical of such early phase studies, the patient cohort showed heterogeneity, but the response indicates that this modality could be a promising option for further investigation.

In contrast to microwave treatment of internal malignancies, which results in tumor ablation through heating, the treatment of skin at low energy levels, as in this study, does not depend on necrosis of skin as epidermal damage was negligible in most cases [16]. The mechanism of action could reflect heat induced cellular stress and induction of apoptotic pathways. But we also consider the possibility of microwave induction of anti‐melanoma immunity. We have previously reported the induction of anti‐HPV immune responses by microwave treatment of cutaneous HPV skin infections mediated by increased MHC class 1 presentation to CD8^+^ T cells [11]. Therefore, the possibility of enhanced CD8 immunity to melanoma induced by microwave is possible.

In this study, RNA‐sequencing was performed in tissue sections to identify biomarkers of response and resistance to microwave therapy, to assess whether treatment upregulates immune‐related genes, and to evaluate potential transcriptional reprogramming of tumor and immune cells that might contribute to sustained tumor control. The early findings here suggest that at a molecular level, complete response is associated with a greater induction of inflammatory responses in microwave treated metastases. The upregulated expression in skin of *CXCL5*, *CXCL8*, *CCL27*, and *IL1A*, which are key mediators of immune cell recruitment, activation, and tumor–immune crosstalk, critical for host immunity, may represent a driver for a cutaneous anti‐melanoma response. This raises the possibility that repeated treatments may be even more effective, possibly by further enhancement of immune signaling, thereby increasing the likelihood of tumor clearance. Expression of melanoma activity markers (*PRAME*, *S100B*, *MLANA*) [17, 18] was reduced in the post treatment samples, which is in line with the clinical observation of reduction in tumor size [16].

When stratifying by therapeutic response, complete responders exhibited a more pronounced suppression of melanoma lineage and oncogenic signaling genes. One of the most prominently down‐regulated genes following microwave therapy was *S100B*, a well‐established melanoma biomarker [18], while *GDF15*, a gene associated with poor melanoma prognosis [19], was also reduced in complete responders alongside other cancer‐promoting genes. In contrast, the gene associated with metabolic and tissue remodeling processes (*FABP4*) was relatively enriched in complete responders [20], suggesting a shift toward tissue restoration and resolution of tumor‐associated signaling. Together these findings indicate that effective treatment response is characterized not only by attenuation of melanoma‐specific transcriptional programs, but also by a reorganization of the tumor microenvironment toward repair and metabolic reprogramming.

The main limitation of this study includes its non‐randomized design and small sample size. Although untreated metastases served as internal controls, the lack of randomization limits the ability to fully separate treatment effects from spontaneous regression or prior therapy. To attempt to control for this risk, we undertook the primary analysis in a pairwise fashion comparing pre‐ and post‐treatment responses in individuals. Inter‐patient variability in disease burden, prior treatments, and immune status may also have influenced response, and the short follow‐up period limits assessment of durability. The cohort consisted of heavily pre‐treated patients with stable melanoma, and referral patterns may have favored more superficial lesions, introducing potential selection bias.

In conclusion, this study suggests that microwave treatment may have potential efficacy in advanced cutaneous melanoma metastases by inducing a shift toward inflammatory and tissue remodeling transcriptional programs and modulating tumor‐associated gene expression. These findings support further investigation to evaluate its role as a potential adjunctive therapy in metastatic melanoma.

## Author Contributions

P.L., D.O., and M.R.A.‐J. wrote the manuscript; D.O., C.O., and M.R.A.‐J. designed the research; D.O., A.V.P., A.W., M.S., and M.R.A.‐J. performed the research; P.L., D.O., A.V.P., M.S., K.B., and M.R.A.‐J. analyzed the data.

## Funding

This study was supported by a Cancer Research UK Centre Network Accelerator grant (C328/A21998), the NIHR Southampton Clinical Research Facility, and generous donation from The Leon Crouch Foundation. Swift device was provided by Emblation Ltd.

## Conflicts of Interest

The authors declare no conflicts of interest.

## Supporting information


**Figure S1:** Differentially expressed genes in post‐treatment samples comparing complete and partial responders.
**Figure S2:** Heatmap of mean expression levels for differentially expressed genes across study groups.
**Table S1:** Clinical characteristics of the study subjects.
**Table S2:** List of DEGs in post‐treatment: a comparison between complete and partial responders.
**Table S3:** List of DEGs in complete responders: a comparison between pre‐ and post‐treatment.
