# Supplementary material for: Transcriptomic Profiling of Cutaneous Melanoma Metastases Treated With Microwave Ablation—Pilot Study
Source: Clin Transl Sci. 2026 Jun 18;19(6):e70638. doi: 10.1111/cts.70638 (PMC13279555; doi:10.1111/cts.70638)
Supplement: Supplementary file 1 — Figure S1: Differentially expressed genes in post‐treatment samples comparing complete and partial responders. Figure S2: Heatmap of mean expression levels for differentially expressed genes across study groups. Table S1: Clinical characteristics of the study subjects. Table S2: List of DEGs in post‐treatment: a comparison between complete and partial responders. Table S3: List of DEGs in complete responders: a comparison between pre‐ and post‐treatment. [file CTS-19-e70638-s001.pdf]

Figure S1. Differentially expressed genes in post-treatment samples comparing complete and partial responders

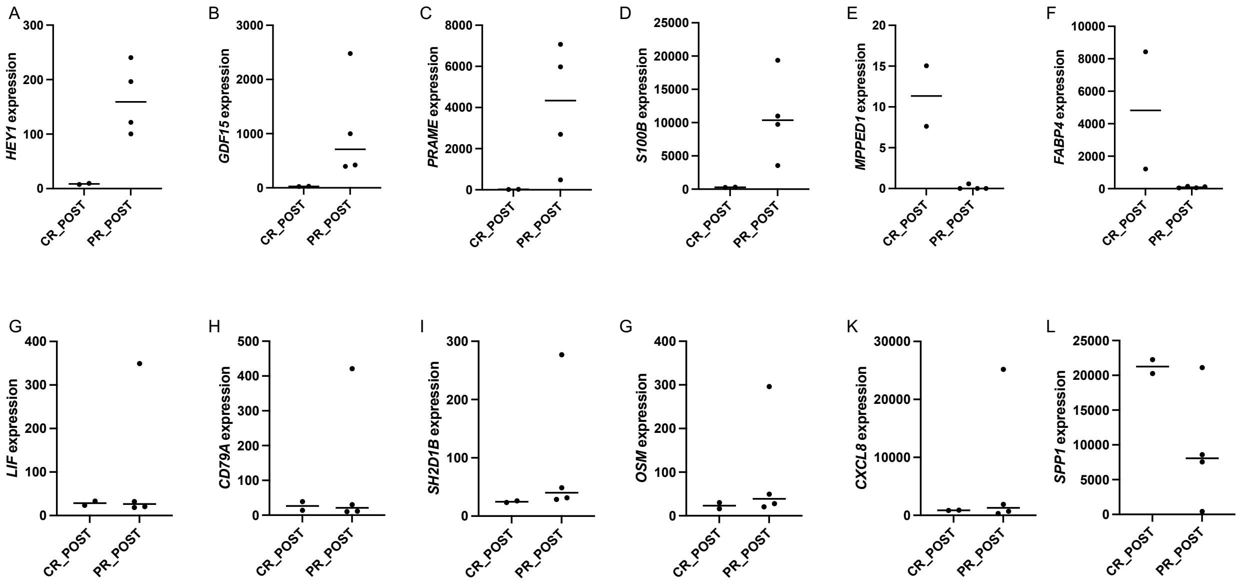

Figure S2. Heatmap of mean expression levels for differentially expressed genes across study groups.

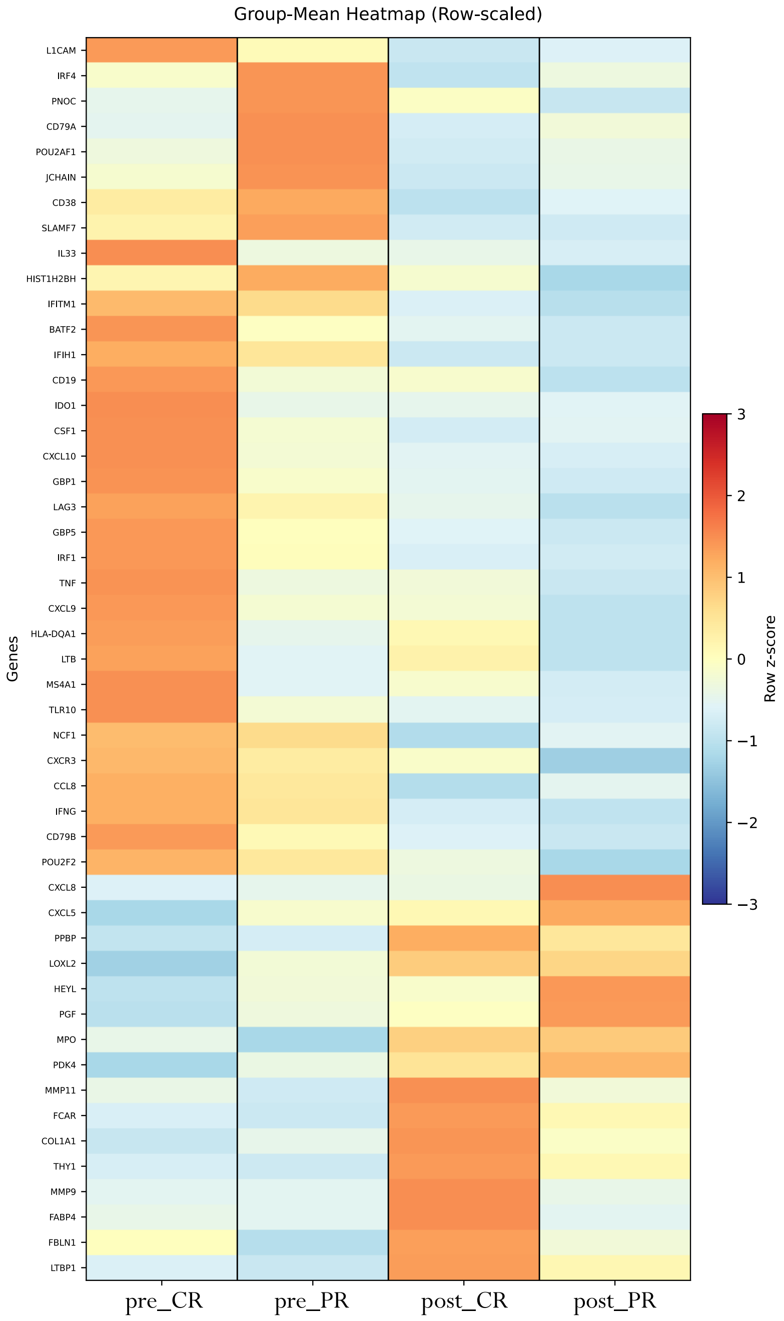

Values are row-scaled and Z-score transformed: red indicates expression above the row mean, while blue indicates expression below the row mean. CR, complete response; PR, partial response.

Table S1. Clinical characteristics of the study subjects

**Patient**	**Age, sex**	**Stage**	**Treatment site**	**Histology, visit 1**	**Post treatment melanoma (T1) clinical status**	**Histology, visit 4**
1	70 F	IIIB	Left achilles	Melanoma	Partial response	Melanoma
2	70 F	IIIC	Left shin	Melanoma	Complete response	No evidence of residual melanoma
3	76 F	IIIC	Left shin	Melanoma	Complete response	No viable melanoma, melanophages only
4	71 M	IIIC	Left calf	Melanoma	Partial response	Melanoma
5	72 M	IVB	Right shin	Melanoma	Partial response	Melanoma
6	57 F	IVA	Right forearm	Melanoma	Complete response	No melanoma, inflammation only
7	74 M	IIID	Left calf	Melanoma	Partial response	Melanoma

Table S2. List of DEGs in post-treatment: a comparison between complete and partial responders

Gene symbol	LogFold change	P value	FDR
**Upregulated genes**			
SPP1	5.657328231	2.14E-05	0.007448103042
FABP4	6.665631431	5.59E-05	0.01104685126
MPPED1	8.02353318	0.00032535563	0.03774125308
**Downregulated genes**			
S100B	-5.314042078	1.22E-06	0.001533183746
PRAME	-4.56367428	2.20E-06	0.001533183746
CXCL8	-4.852927852	1.17E-05	0.005420884507
LIF	-3.622360139	4.14E-05	0.01104685126
HEY1	-3.537827482	6.18E-05	0.01104685126
SH2D1B	-3.482985309	6.35E-05	0.01104685126
OSM	-3.671963108	8.97E-05	0.01387516673
CD79A	-3.987660453	0.0001124533872	0.0156535115
GDF15	-3.961882744	0.0002570259419	0.03252546465

Table S3. List of DEGs in complete responders: a comparison between pre- and post-treatment

Gene symbol	LogFold change	P value	FDR
**Upregulated genes**			
CXCL5	6.03922366	3.16E-09	0.00000241
MMP12	3.30251839	2.65E-06	0.000963912
KRT16	3.38342952	5.64E-06	0.001417831
IL1RN	2.83054424	1.48E-05	0.002014867
KRT17	3.55812378	1.24E-05	0.002014867
MMP9	3.79413011	1.38E-05	0.002014867
DSG3	2.89347157	2.04E-05	0.002224252
IL1A	3.42699432	1.98E-05	0.002224252
CALML3	3.0524958	2.69E-05	0.002663062
CD24	2.56832936	4.04E-05	0.00338836
CCL27	2.53311092	5.82E-05	0.004531609
FABP4	3.89589702	0.00011618	0.008450436
ARG1	3.15756513	0.00022427	0.014392872
SERPINB2	2.66337289	0.00029743	0.017078624
CXCL8	2.13392563	0.0003158	0.017226834
S100A8	2.69719598	0.00054752	0.028445153
BNC1	2.54270738	0.00059363	0.029438559
S100A9	2.67053916	0.00075446	0.034296453
PPBP	2.24638796	0.00084155	0.035312923
SLC2A1	2.08319563	0.00083216	0.035312923
FAM83B	2.11391211	0.00097296	0.039314594
SERPINB5	2.36127672	0.00126687	0.046071898
PDK4	2.0730113	0.0013291	0.046775618
CLCA2	2.07614858	0.00157899	0.049219385
COL1A1	2.35260634	0.00145393	0.049219385
COL3A1	2.28582377	0.00154718	0.049219385
**Downregulated genes**			
PRAME	-6.646220411	4.43E-09	2.41E-06
STK32A	-4.563571157	6.50E-06	0.001417831247
S100B	-4.961540727	3.74E-05	0.003388359721
WARS	-2.567306959	0.0001596473289	0.01088595224
NCF1	-2.335151253	0.0002741871624	0.01661878857
CRISPLD1	-2.761602773	0.0006635011011	0.03147303049
ETV1	-2.274204555	0.001095308179	0.04267790082
ETV4	-2.708416858	0.001222386351	0.04598701753

MLANA	-4.930925136	0.001529651965	0.04921938478
A2M	-2.127665974	0.001637408046	0.04962256051
